# Nature, Causes, and Treatment of Croup

**Published:** 1842-02-12

**Authors:** Caspar Morris


### MEDICAL EXAMINER.

NEW SERIES.

No. 7.] PHILADELPHIA, FEB. 12, 1842. [Vol. I.

### ORIGINAL COMMUNICATIONS.

### *Nature, Causes, and Treatment of Croup.*

By Caspar Morris, M. D.

*Read before the Philadelphia Medical Society, Jan. 29, 1842.*

In the very outset of the inquiry into the nature of croup we are met by the difficulty involved in the question, What is to be understood by the term? In the common language of our patients, sanctioned by the tacit consent of physicians, if not by their direct authority, all cases of disease of the larynx or glottis, accompanied with dry, resonant cough and difficult respiration, are included in this term. The more strict pathologists condemn this nomenclature as indefinite, and assert that in this general group are included no less than three distinct forms of disease, demanding different treatment, depending on diverse causes, and having very different tendencies when left to their uncontrolled course.

It is commonly divided into nervous, spasmodic and inflammatory. Guersent, whose treatise is perhaps one of the best which has been presented to the profession, reduces it still further, and recognises as the only genuine form of croup that in which an exudation of coagulable or plastic lymph takes place in the larynx and trachea, and applies the designation of false croup to the more common and milder disease. I am disposed to think much unnecessary confusion has been created by the attempt to define distinctions where there is no difference. Whoever watches carefully the phenomena of the disease will find, that while peculiar idiosyncrasies, or circumstances beyond our observation, give greater prominence in some cases to the nervous, and in others to the spasmodic symptoms, there are yet to be found in all the traces of inflammatory action in some of its stages and of some form. It is freely admitted that the effusion of the plastic lymph, miscalled a false membrane, is undoubted evidence of the existence of inflammation; but it does not by any means follow that this should be adopted as a pathognomonic sign of pre-existing inflammatory action, yet this is the position assumed by those who assert that there is no croup except where this is present. To ascertain the causes upon which this effusion depends would be an exceedingly interesting subject of examination. Like the other terminations of inflammation, it appears to arise from some unknown condition of the blood at the time. How often do we find it deposited upon ulcers. I have repeatedly, in the earlier periods of my professional career, flattered myself with the belief that an obstinate ulcer had nearly closed, only to be mortified by finding the whole apparent cicatrix peel off like the rind of an orange, proving to have been a mere unorganized deposit; and I have at this very moment a case of fungus of the antrum maxillare under treatment, in which the surface is constantly clothed with a thick deposit, apparently identical with that which has been assumed to be index of real croup. It does not appear to depend, as might be supposed, upon the intensity of the vascular action; thus indicating a *degree* in the scale of inflammation; since there is frequently but little febrile excitement, even in cases which unhappily run on to a fatal termination. Neither can it be assumed to indicate a specific form of inflammation; as it is seen to occur under circumstances so various as forbid such an assumption, in the deposit upon the tonsils of adults, or about the fauces of children, in those formerly mistaken for sloughs upon the surface of ulcers of the leg, and those clothing the elevated surface of redundant growth. Neither does it depend upon a superabundance of fibrine in the blood, since I have never found that which is drawn in croup manifest any especial disposition to the exhibition of the buffy coat.

From the consideration of all these circumstances I have been led to adopt the opinion of the unity of the diseases heretofore separated, and that the apparent difference in the cases is no more than might have been anticipated from the prevalence, in the various individuals subject to it, of some peculiar predisposition to the developement of nervous or spasmodic action, and from some little variety in the part affected. In other words, I should define croup to be an inflammation of the mucous membrane lining the glottis and larynx, attended with more or less spasm of the muscles of the glottis, and frequently terminating in an exudation of plastic lymph. In most cases the force of the disease falls upon the rima glottidis. It instantly provokes a spasmodic action of the muscles of the larynx, by the reflex action the diaphragm. Spasm of the glottis and violent cough are induced, remedies are promptly applied, and the attack is cut short. The symptoms are very analogous to those induced by the introduction of a foreign body, and such as are witnessed continually in the strangulation caused by a crumb being drawn into the orifice of the larynx. In strict language, it cannot be asserted of all these cases that inflammation actually exists; but there is undoubtedly that first step toward it which we are accustomed to designate as irritation, and which, unless relieved, will certainly induce it. The crumb of bread drawn into the orifice of the larynx is a source of irritation, transient in its character, but which, if permanent, would produce all the symptoms of undoubted croup. I have never been so unfortunate as to witness a case in which a foreign body was lodged in the trachea, but the symptoms described are identical with those witnessed in violent cases of spasmodic croup. The attempts to describe the peculiar sound of the cough and inspiration in this disease have been very numerous, and always unsuccessful. There is great difference in different cases. Where spasmodic symptoms predominate, the cough is frequent, violent, and resonant, without any accompaniment that would indicate the least disposition to the formation of mucus in the larynx or glottis. The nostrils are dry also, and the air is drawn into the lungs with great violence, and with a peculiar sound caused by the constriction of the glottis from spasm. In severe cases it may be heard at a great distance. I have distinguished it clearly, in the case of one of my own children, in the yard at a considerable distance from the chamber, though all the doors were closed. This form of the disease generally occurs in the night. The child retires at the usual hour, and without having encountered any unusual exposure, and, which is very singular, is waked by the attack almost uniformly between 10 and 12 o'clock. When it is mild, there is nothing but a dry spasmodic cough, frequently repeated; when more severe the cough is more frequent; still increasing in severity, there is a sense of suffocation greater or less in degree. In my own children, who are all subject to this disease, I have seen the face blackened, the head thrown back, the eyes staring and starting from the sockets, the body bathed in sweat, the hands thrown about as those of a person strangling, and constantly raised to the throat as though by an instinctive effort to remove the obstruction. At other times I have known the same child to suffer for hours from a cough literally incessant, every inspiration being followed by a spasmodic expulsion of the air instead of the natural expiration. Now, all these apparently nervous symptoms are uniformly followed by a catarrh of greater or less severity, with fever, and yield to the remedies usually resorted to for the relief of catarrhal symptoms. The irritation is located at the orifice of the glottis; but let it, instead of this location, develope itself in the larynx and trachea, and instead of these symptoms we have a slight dry cough, with oppression in the breathing, and just in proportion as it invades the glottis is there the exhibition of the symptoms by which it is allied to the cases already described. What it is that causes the termination in the effusion of lymph, rather than the secretion of mucus by which the inflammation is relieved, it is impossible in the present state of our knowledge to explain. This latter, however, is not always a remedial measure; as I remember perfectly to have lost the only son of one of our wealthiest citizens about eight years since, completely suffocated by the quantity of mucus which was poured out into the trachea and bronchial tubes. It was discharged in inconceivable quantities, but still accumulated sufficiently to cause death from suffocation. It would, indeed, be quite as reasonable to elevate into distinct forms of disease the peritonitis or pleuritis terminating in adhesions, effusions, whether bloody or limpid, and purulent secretion, as to contend for the separation of croup terminating in effusion of lymph from that resulting in spontaneous cure or common catarrh. But it is not only from the symptoms which occur in common that we argue for the unity of the disease, but from the fact that the cases are all evidently owing to a common cause. It is during the prevalence of the condition of atmosphere which gives rise to other catarrhal diseases that the various forms of croup are most rife; and the causes which render children liable to the one, give rise to the other. The districts of country in which it prevails are those which are subject to sudden vicissitudes of temperature and especially where there is a prevalence of moisture. Where it has prevailed, as an endemic, it has been associated with the other forms of catarrh in adults. Nor must it be forgotten that in two symptoms which have by many authors been thought peculiar, it is also allied to the catarrhal affections. I allude to its paroxysmal attacks and diurnal exacerbations, which are very distinctly marked. To sum up all that has been said of its nature, it is a catarrh which, instead of diffusing itself over the whole mucous membrane of the air passages, expends its force upon the upper portion, producing, as the case may be, and from unknown causes, spasm of the glottis with hoarseness and cough; strangulation, or loss of voice; cough and suffocation, the causes which determine these variations being as unknown as those which lead individuals exposed to malaria to have intermittent or remittent fevers.

The treatment of croup is as various as the ideas on the nature of the disease. Musk and assafœtida, by the advocates of a nervous origin; bleeding by those who look exclusively to inflammation; emetics and expectorants and mercurials by others. In this, as in all other cases, the eclectic practitioner will be most successful. He will find occasion in some instances to resort to nearly all these means in the progress of the case, and more will depend upon the judgment with which they are applied, both as to time and degree, than upon the remedies themselves. The best remedy may not only miss of good effect, but prove wholly baneful if unskilfully used. In the most common form of the disease, that designated by the French nosologists "false croup," known by some as "Millar's asthma," but, as I believe, only a peculiar modification of laryngeal inflammation, in which the symptoms of spasm predominate, a nauseant will almost always afford relief if promptly resorted to. My own family of five children are all liable to these attacks, and my habit is to give a dose of the compound syrup of squills sufficient to produce a little nausea, but not enough to vomit immediately on the occurrence of the first cough. Should this not be sufficient to check it entirely, the dose is repeated, still short of an emetic; by this course I have for several years past averted the violence of the attacks, which were frequently repeated during each winter, when my family was much smaller. The next step, when these prove insufficient, is to resort to a warm bath, which, acting both as a revulsive and relaxant, confirms the impression previously made by the syrup. Should this, however, fail to remove the irritation, there is every reason to fear that it is passing the stage of mere vascular engorgement into that of acute inflammation, and it becomes necessary to resort to more energetic measures. The compound syrup of squills may be advantageously exchanged for a solution of tartrate of antimony or sulphate of zinc, which in many cases is better than the antimony on account of its being less liable to produce the extreme depression which sometimes follows the use of the former medicine. Indeed I have known instances in which death has followed the injudicious use of the antimony, and have been myself consulted in one case in which the life of the child was saved with some difficulty. The well known fact that the susceptibility of the stomach to the impression of emetics is diminished by this disease is thought to justify the exhibition of immoderate doses, while the apparent violence of the disease, seeming to call for very prompt measures, causes the friends to reiterate the doses at too short intervals. This is, indeed, a disease which demands from the physician personal attention. If it passes into the stage now under consideration he should not confide the administration of the remedies to either parents or nurses, but should watch their effects and graduate the doses and apply them with his own hands. During the use of the emetics and warm bath much advantage may be received from the use of external irritation. Mustard poultices may be applied to the neck and chest, both back and front, with great advantage, or the spirits of turpentine may be applied on flannel cloths, producing a very prompt rubefacient effect. Should these means fail of subduing the disease, or should the emetics given in reasonable doses not produce their specific effects, a resort to depletion becomes necessary, and will be indicated in the great majority of cases by the vascular excitement which occurs. Considerable difference of opinion prevails as to the best mode of effecting this reduction; some advocating the application of leeches or cups; some direct depletion from the external jugular vein; and others drawing it in the usual manner from the veins of the arm. This is, I think, on every account the most advisable. The object is to make a prompt and decided impression on both the vascular and nervous systems. This is certainly induced more readily by the abstraction of a given quantity of blood from the great vessels than from the capillary system. This point has been so often argued on this floor, that it is hardly needful to go over the ground already well beaten. Should there be any great difficulty in opening a vein in the arm, I should not hesitate to resort to the external jugular, though it is only in such circumstances I should think it justifiable, on account of the needless soiling of the child, and the alarm necessarily caused by an unusual operation. There can be no possible advantage to the patient over a common venesection. The quantity of blood taken should never be great. The experience of fifteen years, and some of it of a sorrowful kind, has convinced me fully that children bear bleeding *illy*, and in asserting my conviction that as many children have sunk under the ill-judged use of depletion as from incurable disease, I would not cast censure upon others without taking to myself a full share of it.

We should not lay aside the use of emetic articles; and in this stage they should be simple emetics. The tartrate of antimony is that in which I am disposed to place most confidence. It acts undoubtedly in the same manner as in pneumonia, where its beneficial influence is so universally recognised. Some of the German authors are disposed to claim great superiority for the preparation of copper; but apart from their violence and uncertainty, there is more difficulty both in procuring them and in getting children to take them. The antimony, being tasteless, may be given without difficulty, but the caution before given should never be lost sight of, and the effect should be watched from hour to hour. Should further depletion be deemed proper, it may be effected by leeches to the neck or upper part of the sternum; but the effect of these should also be watched carefully, as one case of death occurred in my practice by the hæmorrhage from Spanish leeches. The child, apparently relieved, was supposed to be sleeping, and left undisturbed until the morning, when it was found to be dead, its under garments and bed saturated with blood; and one of my own children narrowly escaped death from the same cause. Should these remedies not subdue the disease, it will be found to have changed its character; the violent spasmodic cough will have been exchanged for one dry, husky, and occasional; there will be permanent difficulty of breathing, aggravated in frequently repeated paroxysms; and the voice will have exchanged its roughness for a peculiar whistling whisper. When these changes occur, there is great reason for alarm; and though the symptoms enumerated may all exist without there being necessarily an exudation of lymph, it is proper to suspect it, and make a cautious examination of the fauces. There will often be found a coating, more or less dense, spreading over the lateral or posterior part of the pharynx or the tonsils, sometimes merely a slight milky pellicle, and at others a dense white deposit, sometimes nearly a line in thickness. Various means have been resorted to in the hope of detaching this membrane, which has been erroneously thought to occasion death by a mechanical closure of the glottis. The application of the nitrate of silver, either in solution or the solid stick, was at one time suggested and freely tried, but without success, and mechanical efforts have even been resorted to; it need not be added, with fatal results. It seems difficult to believe that the celebrated Dupuytren should have ventured on such an experiment; but it is reported by good authority that in one case he passed a sponge attached to a flexible handle into the larynx of a child, with the hope of wiping it out as a gunner would sponge his piece!—it need hardly be added that the child died instantly in his hands. Even admitting for a moment that such rude measures should be successful in the removal of the deposit, it would doubtless be immediately renewed, and, in all probability, with increased thickness, as the inflammation upon which it depends could not fail to be aggravated by the ill-judged efforts of the practitioner. There has been too much disposition to regard this effusion as the disease itself, and not as a consequence merely, which it is as surely as the mucous discharge from the nostrils or the bronchial tubes themselves is but a result of the inflamed state of the membrane. The nostrils may be cleansed for convenience, and mucous coughed up for comfort, but the choryza or bronchitis still continues. So, even if we could certainly evacuate the whole effusion, whether mechanically or by emesis, still our patients would derive but temporary relief unless we at the same time removed the diseased state of the membrane itself. I therefore persevere, in this stage of the disease, in the use of emetic articles, not only, nor even so much, for their mechanical as their constitutional effect. They are antiphlogistic in their action on the system generally. With the same view mercurials may be resorted to, and should be used freely, as it is important to produce their specific impression as promptly as may be. Calomel should be given internally; and in severe cases, frictions with mercurial ointment, may be tried with propriety. I am satisfied I have seen great benefit result from the use of the nitrate of potass also, and resort with more confidence to a combination of calomel, nitre and tartrate of antimony, after I have pushed the bleeding and vomiting so far as I think prudent, than to any other measures. I adopted this treatment from my views of the pathology of the disease, and experience has fully sustained it. The only cases I have seen recover, in which there was reason to suspect the exudation of coagulable lymph, have been relieved by this treatment; and the only cases that have died, have, I believe, owed their fatal termination either to the negligence of the parents in allowing the disease to progress too far before calling in aid, or to the too free depletion employed, perhaps at too late a stage of the disease.

The calomel treatment has undoubtedly been abused in some hands. There are cases on record in which 50, 60 or 100 grains have been given to young infants in the course of a few hours. I had myself an interesting case a few years since in which a child had been permitted to pass the whole night in violent croup, without any effort being made to relieve it. I was called to it about daylight in the morning; bled it, leeched it, gave it emetics, blistered it, without affording relief. An eminent practitioner and professor was called in consultation, who suggested the employment of calomel in doses of ten grains, every hour, (the infant was under two years of age.) Having exhausted my own armory, I consented to resort to his, only upon the condition that he should assume the entire responsibility, as it was the child of a gentleman occupying a prominent position in society, and feeling confident that if it died the fatal result would be attributed to the remedy rather than the disease. When we commenced the use of the calomel the skin of the little patient was cold and bathed in perspiration; the breathing laborious; the cough dry and husky; the voice whispering; restlessness extreme. It soon became quiet, with a warm skin and a cessation of the cough; but within 12 hours, after having taken the 10th dose, it was seized with convulsions and died. Might not a more moderate use of the calomel have produced a more favourable result? How far may the convulsions have been owing to gastric irritation produced by the large quantities of the drug? These are questions which have often presented themselves to my mind during the six years which have elapsed since the case occurred. Whilst this constitutional treatment is pursued, the propriety of aiding it by external remedies will present itself to every mind. This is, indeed, an essential part of the treatment, and can no more be dispensed with than any that has been mentioned; but of what kind shall it be? Stimulating baths and frictions—the application of dry heat of a sufficient degree to be decidedly counterirritating, are, I think, all that are expedient. The coldness of the surface, especially in the latter stage, and the evident congestion of the lungs and brain, clearly demand some such expedients; and my personal experience with vesications, of whatever kind, is so painful, that I think I may say I will never voluntarily blister an infant in any form of disease. I am sure that I have lost at least half a dozen children from the effect of blisters alone. They have either sloughed or become exceedingly irritable, spreading ulcers, and have worn out children already wasted by disease and depletion, who might have surmounted the disease alone, but have sunk under the remedies; and yet I have never failed to direct them to be removed so soon as the least redness of the skin is perceptible, which is mostly in about two hours. A soft poultice applied upon the removal of the cantharides plaster at this period causes free vesication, and with much less pain than is incident to the sore made by the longer continuance of the blistering ointment. But to return to the mercurial treatment, the object with which it is employed is to promote the resolution of the inflammation by its specific effect, just as we use it in any other inflammation of the mucous tissue; dysentery and diarrhœa, for example; which are closely allied in character to the disease under consideration. It is no uncommon thing to see large quantities of flocculent lymph discharged in cases of dysentery; and I have been gravely assured by intelligent parents that their children have passed away a part of the intestines, when, upon examination of the stool, it has proved to be only a mould of a part of the rectum formed of this lymph.

There is one other class of remedies which I have undoubtedly seen useful. These are the alkaline subcarbonates. I have long been in the habit of adding them to expectorant mixtures, and with manifest advantage. It is, moreover, asserted by good authors that they exercise a direct solvent power on the exuded matter. These are the leading features of the treatment in this disease, so violent and rapid in its course and so often fatal in its termination. What is called its third stage is, in fact, but the commencement of the moribund condition. The lungs and brain become congested; the blood ceases to be arterialized; the respiration becomes more laborious; the surface cold, bathed in perspiration, and pallid; the countenance livid. Yet even here we should not despair, as there are well authenticated instances in which the patient has revived under the most desperate circumstances. An old and intelligent lady assured me that in the case of one of her children she had thrust her fingers into the mouth and withdrawn a well formed tube from the larynx, even when the child was supposed to be moribund, and as such abandoned by one of our most eminent practitioners, and it recovered. It is in this stage that the disease presents its most appalling features. The mind unimpaired; the countenance expressive of the most intense anguish; the child speechless, yet imploring with "mute eloquence" the aid of every one approaching; unable to lie down or to maintain the same position for a moment; no spectacle can be imagined more appalling. The stimulating expectorants and diffusible stimuli must here be our dependance; gum ammoniac, assafœtida, but especially carb. ammon. and wine whey; with these may be joined frictions with ammoniated linament or ol. succini. The objection made by some on the score of injury thought to result from their inhalation, is more theoretical than practical. It is not to be wondered at that tracheotomy should have been thought of in such cases, and it would have been unpardonable not to have given it a trial. In this city it has been uniformly without success. In one case Dr. Rhea Barton, at the earnest solicitation of the physicians and friends, performed the operation when the child was apparently *in articulo mortis.* The first access of air to the lungs produced effects as wonderful as those of Galvanism. What had been apparently a corpse, looked aound on its parents, embraced them, drank, and I believe talked. It soon, however, sank again into the stupor of death, and crushed the hopes which had been so highly excited. By the latest accounts the numerical method has been brought to bear upon this point, and the result affords but little encouragement.

Of 140 cases reported by French operators, 112 resulted in death, aud 28 recovered. Of these 28, 20 were in the practice of Trousseau; and I believe I only represent the general feeling of the profession in this city in asserting that many of his operations have been performed upon cases in which it was by no means necessary. If the mechanical obstruction of the glottis was the cause of death, there could be no hesitation in resorting to tracheotomy in every case which threatened a fatal result. But I am satisfied this is by no means the case; on the contrary, the cough generally removes this obstruction from the upper part of the larynx, even in those cases where it still adheres throughout the trachea and bronchial tubes.

